# Heat dissipation in Sm^3+^ and Zn^2+^ co-substituted magnetite (Zn_0.1_Sm_x_Fe_2.9-x_O_4_) nanoparticles coated with citric acid and pluronic F127 for hyperthermia application

**DOI:** 10.1038/s41598-021-96238-2

**Published:** 2021-08-18

**Authors:** S. Shatooti, M. Mozaffari, G. Reiter, D. Zahn, S. Dutz

**Affiliations:** 1 Faculty of Physics, University of Isfahan, 81746-73441 Isfahan, Iran; 2 Institute of Physics, University of Freiburg, Hermann-Herder-Straße 3, 79104 Freiburg, Germany; 3 Institute of Biomedical Engineering and Informatics (BMTI), Technische Universität Ilmenau, Gustav-Kirchhoff-Straße 2, 98693 Ilmenau, Germany

**Keywords:** Materials science, Nanoscience and technology, Physics

## Abstract

In this work, Sm^3+^ and Zn^2+^ co-substituted magnetite Zn_0.1_Sm_x_Fe_2.9-x_O_4_ (x = 0.0, 0.01, 0.02, 0.03, 0.04 and 0.05) nanoparticles, have been prepared via co-precipitation method and were electrostatically and sterically stabilized by citric acid and pluronic F127 coatings. The coated nanoparticles were well dispersed in an aqueous solution (pH 5.5). Magnetic and structural properties of the nanoparticles and their ferrofluids were studied by different methods. XRD studies illustrated that all as-prepared nanoparticles have a single phase spinel structure, with lattice constants affected by samarium cations substitution. The temperature dependence of the magnetization showed that Curie temperatures of the uncoated samples monotonically increased from 430 to 480 °C as Sm^3+^ content increased, due to increase in A-B super-exchange interactions. Room temperature magnetic measurements exhibited a decrease in saturation magnetization of the uncoated samples from 98.8 to 71.9 emu/g as the Sm^3+^ content increased, which is attributed to substitution of Sm^3+^ (1.5 µB) ions for Fe^3+^ (5 µB) ones in B sublattices. FTIR spectra confirmed that Sm^3+^ substituted Zn_0.1_Sm_x_Fe_2.9-x_O_4_ nanoparticles were coated with both citric acid and pluronic F127 properly. The mean particle size of the coated nanoparticles was 40 nm. Calorimetric measurements showed that the maximum SLP and ILP values obtained for Sm^3+^ substituted nanoparticles were 259 W/g and 3.49 nHm^2^/kg (1.08 mg/ml, measured at f = 290 kHz and H = 16kA/m), respectively, that are related to the sample with x = 0.01. Magnetic measurements revealed coercivity, which indicated that hysteresis loss may represent a substantial portion in heat generation. Our results show that these ferrofluids are potential candidates for magnetic hyperthermia applications.

## Introduction

In diagnostic and therapeutic applications, magnetic nanoparticles (MNPs) and their biocompatible ferrofluids are receiving increased attention due to their heat dissipation and magnetic tracer properties under an external AC magnetic field^1,2^. Due to their unique and novel properties, such as high electrical resistivity, magnetic and optical properties, low eddy current and low dielectric losses, MNPs based on iron-oxides and their rare earth substituted analogs have been intensively investigated in biomedical applications^3–5^.

Rare earth ions have unpaired 4f. electrons, which are the source of magnetocrystalline anisotropy due to their orbital shape and strong spin–orbit coupling^4,6^. Rezlescu et al. reported a significant alteration of electromagnetic properties of ferrites by replacing a small amount of rare earth ions for Fe^3+^ ions. Such substitution resulted in a strain, which induced structural distortions and increased the ferrite's resistance to oxidation^7^. Because of the large radius of rare earth ions, they have a limited solubility in the spinel ferrite lattice. Therefore, just a few of the Fe^3+^ ions are substitutable by rare earth ions in the spinel structure^8^.

Due to their remarkable effects on structure and magnetic properties of ferrites, samarium cations and/or other rare earth ones, have been investigated as substituting ions in spinel ferrite. It has been shown in several studies that substitution of iron ion by samarium ion in spinel ferrites has not the same effects on magnetic and structure properties as other rare earth ions have^9–11^. Nabeel showed that samarium complexes indicate higher anticancer activities than other tested rare-earth (lanthanide) ones^12^.

MNPs play an important role as heat exchangers in a ferrofluid in the RF magnetic hyperthermia. Use of MNPs causes fewer side effects as damage to healthy tissues than normal cancer treatment methods. This method decreases the cancerous cells and augments their sensitivity to chemotherapy and radiation additionally by rising the local temperature of targeted tissues to an interval from 42 to 46 °C^13,14^. The heating efficiency of MNPs is measured through the specific loss power (SLP) that is also referred to as specific absorption rate (SAR). It has been found that the SLP of MNPs can be modified by tuning saturation magnetization, effective anisotropy, and particle size^15^.

MNPs tend to agglomerate because of their large surface to volume ratio and magnetic dipole–dipole interactions^1^. In order to reduce and/or avoid sedimentation and to enhance biocompatibility and functionalization, it is necessary to coat MNPs with surfactants or polymers. The stability of ferrofluids against agglomeration is related to a competition between various interactions, such as Van der Waals, magnetic dipole–dipole interactions, viscous drag force from the carrier fluid, and electrostatic and steric repulsion resulting from the surfactants in the coating^16,17^.

Achieving stability of ferrofluids in polar media (e.g., water) is a more complicated challenge than non-polar media (e.g., oil). Electrostatic repulsive forces between MNPs due to a high electric surface charge density may allow achieving long-term stability for water-based ferrofluids. Steric stabilization may not be sufficient for obtaining a stable colloidal suspension of particles with large magnetic core which introduce strong magnetic attraction forces. In such cases, strong electrostatic repulsion forces between the particles can be obtained by coating them with a highly charged material. To this end, citric acid (CA) is an appropriate candidate to coat particles, specifically for magnetic nanoparticle with strong magnetic interaction. CA has a high biocompatibility and introduces both, electrostatic and steric repulsion effects. CA has three carboxyl groups and a hydroxyl group that chemisorb to the iron oxide surface of the nanoparticles by forming a carboxylate complex with the Fe ions^17–19^.

Pluronic F127 is a biocompatible triblock polymer of amphiphilic nature which is composed of two hydrophilic chains of polyethylene oxide (PEO) and a hydrophobic chain of polypropylene oxide (PPO)^20^. At low temperatures and/or low concentrations in aqueous solution, PEO-PPO-PEO copolymers are present as individual unimers. By increasing copolymer concentration and/or solution temperature, thermodynamically stable micelles are formed. The corresponding critical micelle concentration (CMC) is temperature dependent^21^. To stabilize magnetic nanoparticles by coating them with pluronic F127 as used for magnetic diagnostic (i.e. magnetic resonance imaging (MRI)) and therapy (i.e. magnetic hyperthermia), pluronic F127 is normally accompanied by oleic acid and/or other polymers more than CA or in addition to CA^22,23^.

In this work, due to high biocompatibility of Zn^2+^ and anticancer activity of samarium complexes ^12,24^, Sm^3+^ and Zn^2+^ co-substituted magnetite Zn_0.1_Sm_x_Fe_2.9-x_O_4_ (x = 0.0, 0.01, 0.02, 0.03, 0.04 and 0.05) MNPs, were synthesized via co-precipitation route at 80 °C. In a simple process, these MNPs were coated by CA and pluronic F127, respectively. The effect of substitution of iron by Sm^3+^ ions on the physical properties of the uncoated nanoparticles and their ferrofluids were studied.

Reduction of drug dose in all medical treatments is a goal. It is desirable to attain the target temperature in MH with as small amount of MNPs as possible to be delivered in tumors, which needs SLPs as high as possible^17^. Also synthesis of stable suspensions of large (d > 20 nm) magnetic core–shell nanostructures is another advantage^25^, which achieved in this work. Additionally maximum specific absorption rate (259 W/g) and intrinsic loss power (3.49 nHm^2^/kg) were achieved for aqueous ferrofluids, which were related to the sample with x = 0.01, in a concentration as low as 1.08 mg/ml.

## Materials and methods

### Preparation of uncoated and coated Sm^3+^ substituted Zn_0.1_Sm_x_Fe_2.9-x_O_4_ nanoparticles

**Figure 1:**
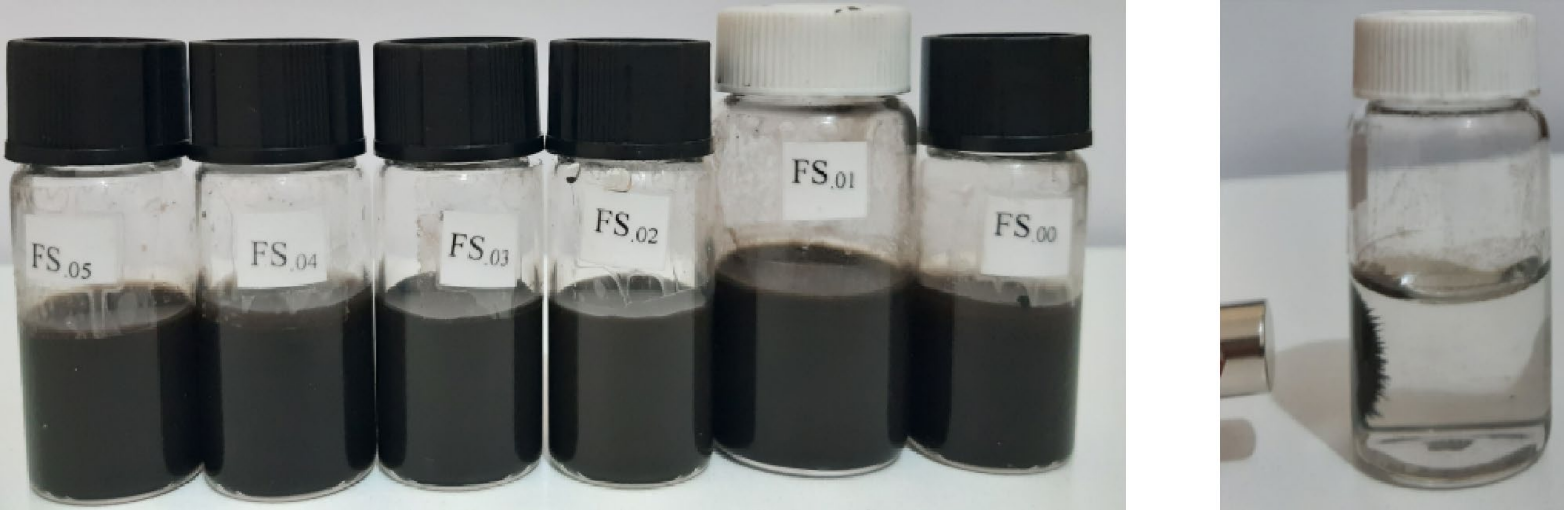
Images of the MNP@CA@Pluronic ferrofluid samples (left) and also showing attraction towards a magnet (right).

**Figure 2:**
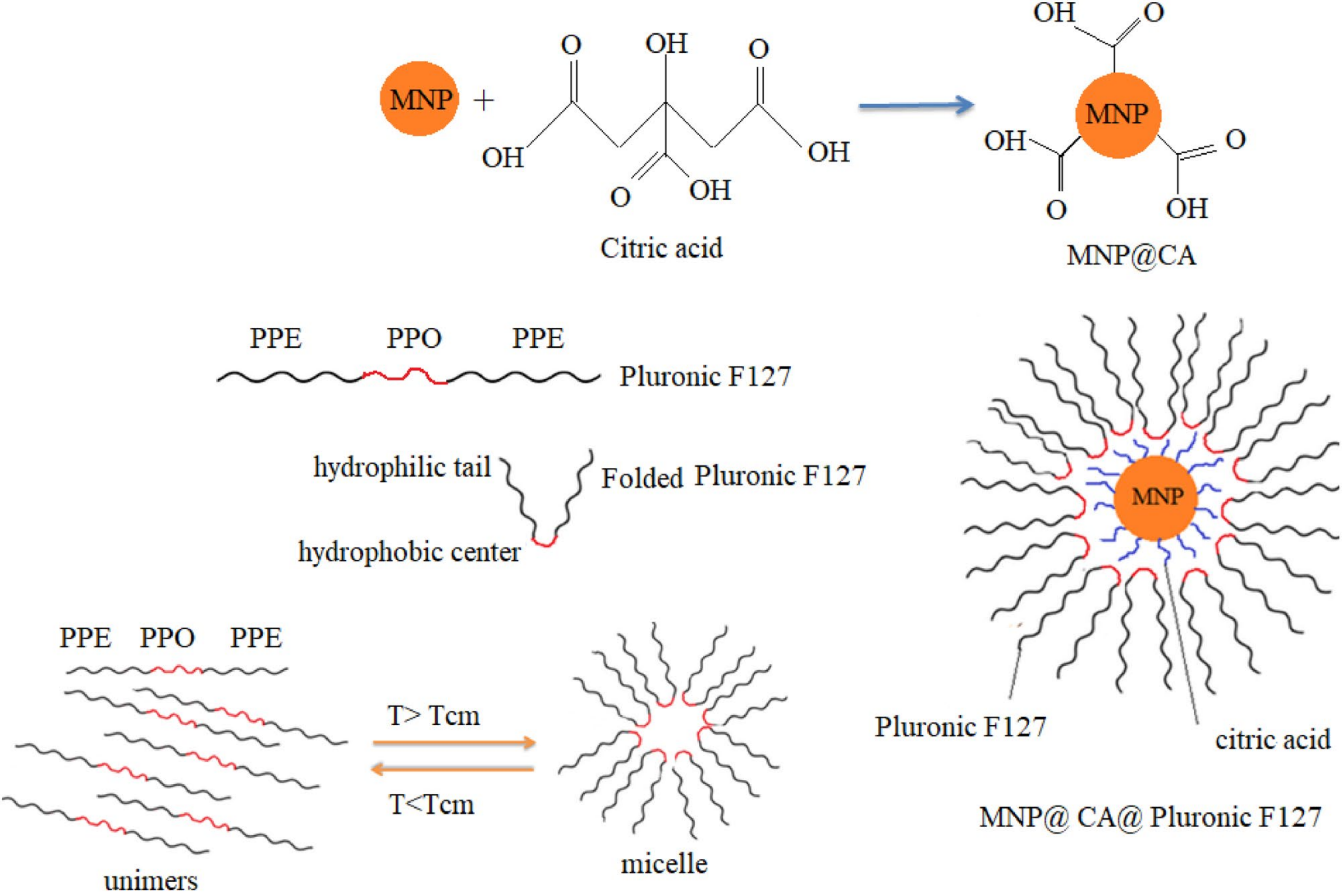
Schematic illustration of the coating process applied to the nanoparticles.

Sm^3+^ substituted Zn_0.1_Sm_x_Fe_2.9-x_O_4_ (x = 0.0, 0.01, 0.02, 0.03, 0.04 and 0.05) nanoparticles were prepared by co-precipitation method at 80 °C and were coated with citric acid and subsequently coated with biocompatible copolymer pluronic F127. The reagents, including FeSO_4_.7H_2_O and Zn (NO_3_)_2_.6H_2_O, with minimum purities of 99% were purchased from Merck Co., Germany, citric acid (C_6_H_8_O_7_) with a minimum purity of 99.5% was obtained from Carl Roth GmbH + Co.KG., Germany, NaOH flakes with a minimum purity of 98%, Sm (NO_3_)_3_.6H_2_O with a minimum purity of 99% was provided from Alpha Acer Co., and Pluronic F127 was obtained from Sigma Aldrich Co., USA. For each sample, proper stoichiometric ratios of FeSO_4_.7H_2_O (1 M), Zn (NO_3_)_2_.6H_2_O (1 M) and Sm (NO_3_)_3_.6H_2_O (1 M) were separately dissolved in deionized double distilled water and stirred on a magnetic stirrer at room temperature to get clear solutions, which were then mixed together (40 ml). In this way, 40 ml of 3 M NaOH solution was added into each mixture abruptly. All obtained precipitates were dark green and were heated and stirred on a magnetic stirrer at 80 °C till their hues changed to black. The dark green precipitates were included iron (II), zinc and samarium compositions, which some Fe^2+^ ions were partially oxidized to Fe^3+^ in presence of air oxygen by oxidizing solution. These compositions formed via separate reactions occur during the formation of Sm-Zn co-substituted magnetite nanoparticles. The obtained black precipitates were magnetic and responded to an NdFeB permanent magnet strongly. The precipitates were decanted magnetically and were washed with deionized double distilled water several times to eliminate excess ions and get a neutral pH. For powder characterization, a small amount of each washed precipitate was dried in air at room temperature. These samples were named S_.00_, S_.01_, S_.02_, S_.03_, S_.04_ and S_.05_ for x = 0.0, 0.01, 0.02, 0.03, 0.04 and 0.05, respectively. The nanoparticles were coated to reduce their toxicity and to modify their surface. Approximately 2 g of each of the washed as-precipitated samples were dispersed in 300 mL milli-Q water and sonicated for 15 min, using a FRITSCH ultrasonic bath. A CA solution (1.7 g in 25 ml of milli-Q water) was added to each solution and finally each mixture was stirred further for another 10 min. Each mixture was heated and stirred at 80 °C for 90 min, and then cooled to room temperature freely. The coated samples were decanted and washed with milli-Q water 4 times to get ferrofluids. They were labeled with S_.00_ @CA, S_.01_@CA, S_.02_@CA, S_.03_@CA, S_.04_@CA and S_.05_@CA for x = 0.0, 0.01, 0.02, 0.03, 0.04 and 0.05, respectively. A solution of pluronic F127 (1.5 g in 20 ml of milli-Q water) was prepared at temperatures below 40 °C. This solution was added to each ferrofluid (coated nanoparticles were dispersed in 200 mL milli-Q water) and then vigorously stirred for 2 h on a hot plate while the temperature of each solution was gradually increased up to 39 °C. The samples were washed with milli-Q water 3 times to remove excess polymer and were labeled with FS_.00_, FS_.01_, FS_.02_, FS_.03_, FS_.04_ and FS_.05_ for x = 0.0, 0.01, 0.02, 0.03, 0.04 and 0.05, respectively. According to the data obtained from the different analyses on the samples, although the nanoparticles in slurries were large in size and had strong magnetic interactions, the ferrofluids were stable in an aqueous environment (pH 5.5). Stabilization might be related to the provided thermal energy and due to steric repulsive through the polymer coating. The PPO part of the pluronic F127 adsorbed on the surfaces of the nanoparticles. The PEO parts formed a water soluble shell around the particles, generating a repulsive force for entropy reasons. Along with the CMC, we also expect that the solubility was temperature dependent. At high temperatures the value of the CMC is normally higher than at low temperatures^21,26^. Images of the ferrofluids and a schematic representation of the coating process are presented in Figs. 1 and 2, respectively.

The XRD patterns of the samples were taken at room temperature using an X-ray diffractometer (Philips, X'PERT model), with Cu-Kα radiation (λ = 1.5406 Å), at a scanning rate of 0.04° per 1 s, and their full Rietveld-refined patterns were fitted by the MAUD program. The lattice constants of the uncoated nanoparticles were calculated by least-squares method^27^. The mean crystallite sizes of the nanoparticles were estimated from the broadening of the XRD peaks, using Scherrer's formula, D = 0.9λ/β cos(θ), where D is the mean crystallite size, λ is the used X-ray wavelength, θ is the Bragg angle and β is the full width at half-maximum (FWHM) intensity of the (311) peak, respectively.

The morphology, particle size and size distribution of coated samples were studied using a transmission electron microscope (JEOL JEM-2100F model). The mean particle size from TEM images was calculated by Image J software. The colloidal properties of the nanoparticles in aqueous suspension (mean hydrodynamic size and polydispersity index (PDI)) were studied by dynamic light scattering (DLS, Horiba Scientific) at pH 5.5 and T = 25 °C. Fourier transform infrared (FTIR) spectra were recorded with a Jasco spectrometer (6300 model) between 4000 and 400 cm^−1^. The Curie temperature of the samples was determined by DTG/M/method^28^ in a weak magnetic static magnetic field. In our previous study, we elucidated the Curie temperature measurements^29^. Magnetic measurements were performed at room temperature, using a vibrating sample magnetometer (VSM) (Lake Shore Cryotronics, 7407 model) with a maximum applied magnetic field of ± 18 kOe.

**Figure 3:**
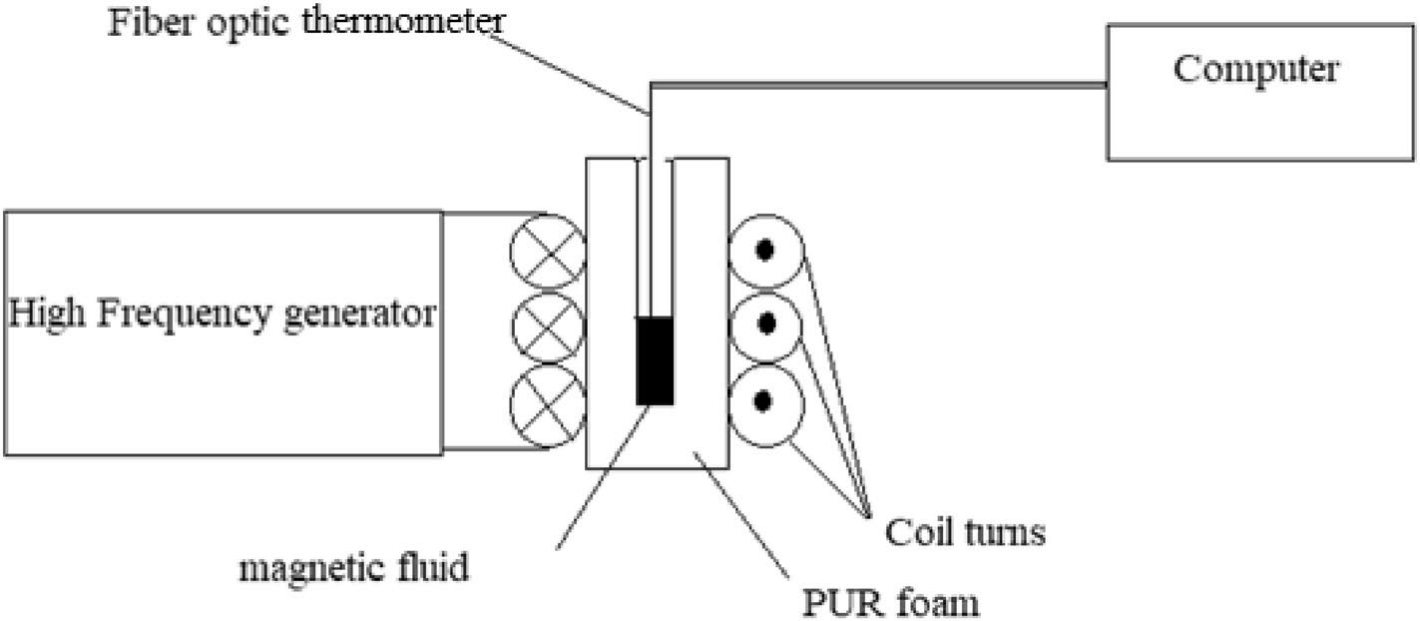
Scheme of the set up used for the measurement of the specific loss power.

To determine heating efficiency, the initial temperature slope dT/dt, an alternating magnetic field was generated in an induction coil, using a high-frequency induction machine (EFD Induction, Germany) (Fig. 3). The water-cooled coil was made of copper tube which has 3 turns and a mean diameter of 5 cm. The rms field strength and the field frequency were 16 kA/m and 290 kHz, respectively. The temperature was measured with a fiber optic thermometer with precision of 0.1 °C (FOtemp, OPTOcon, Germany) and the probe was kept in the center of the ferrofluid (Fig. 3). The nanoparticles suspensions (a 0.5 ml of ferrofluid put in a 2 ml Cryovial) were thermally isolated with polyurethane foam and placed at the center of the copper coil. The heat efficiency related to the specific loss power (SLP) defined as heat power dissipation per unit mass of nanoparticles, is expressed by:

1 $${\text{SLP}}\;{\text{(W/g)}} = {\text{dT}}/{\text{dt}} \times \Sigma {\text{m}}_{{\text{i}}} {\text{C}}_{{\text{i}}} /{\text{m}},$$

where m_i_ and C_i_ are the mass and specific heat capacity of each component of the magnetic ferrofluid, and m is the mass of the magnetic nanoparticles^30^. Specific heat capacities of water and magnetite are 4180 and 937 J/kg K, respectively^31^. The specific heat capacity of water was used for aqueous ferrofluids with a particle concentration lower than 2% in the suspension^30^. Since SLP is measured for different magnetic field strengths and frequencies and in different laboratories, the intrinsic loss power (ILP) is determined by using the following equation^32^:

2 $${\text{ILP}}\;\left( {{\text{nHm}}^{2} {\text{kg}}^{ - 1} } \right) = {\text{SLP/H}}^{2} {\text{f}}$$

## Results and discussion

### Structural characterization

**Figure 4:**
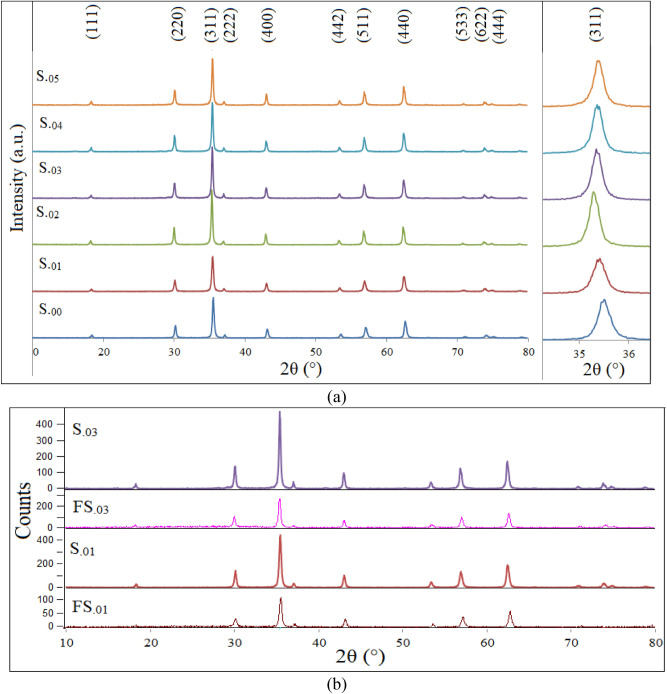
(**a**) XRD patterns of the as-prepared nanoparticles. Inset exhibits (311) diffraction peak to show the displacement of the peaks as Sm^3+^ content varies, and (**b**) XRD patterns of selected uncoated and coated nanoparticles.

**Figure 5:**
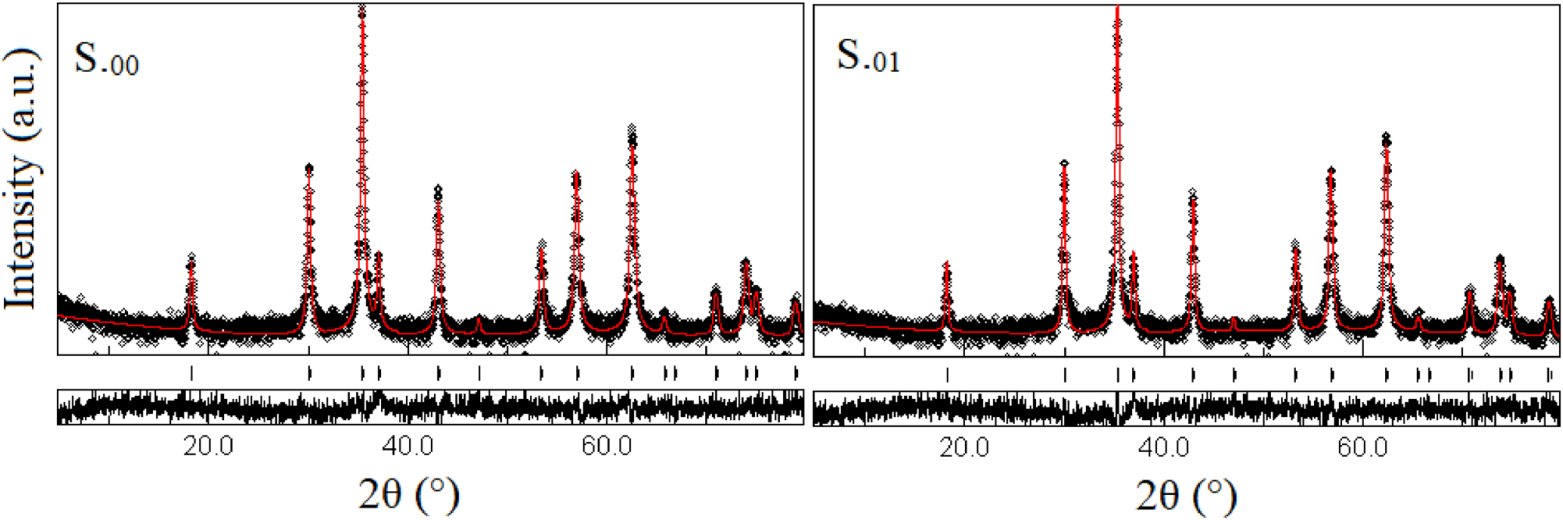
The Rietveld-refined XRD patterns of as-prepared specimens as marked on the patterns.

Figure 4a indicates XRD patterns of the as-prepared Zn_0.1_Sm_x_Fe_2.9-x_O_4_ (x = 0.0, 0.01, 0.02, 0.03, 0.04 and 0.05) nanoparticles. As can be seen, all labeled main diffraction peaks were fully consistent with the cubic spinel structure (PDF card No.: 01-089-1009), indicating that all samples are single phase. Also it can be seen that, by increasing the Sm^3+^ content, the main diffraction peaks were initially shifted to lower diffraction angles, which is due to, based on Vegard's law^33^, to replacement of smaller Fe^3+^ (0.0645 nm) ions by larger Sm^3+^ (0.096 nm) ions. The radii of Fe^2+^ (0.063 and 0.078 nm), Fe^3+^ (0.049 and 0.0645 nm), Zn^2+^ (0.06 and 0.074 nm) and Sm^3+^ (- and 0.096 nm), which the first and second values referred to those of (tetrahedral (A) and octahedral (B) sites), respectively^34,35^. The site preferences of Sm^3+^ and Zn^2+^ ions are octahedral and tetrahedral sites, respectively. By increasing the Sm^3+^ ion content, the main XRD diffraction peaks were shifted to larger angles, due to either internal strains which can be responsible for both increase and decrease of lattice parameters^36,37^ or due to redistribution of cations between A and B sites in order to relax the strain^38^. As the expansion of a lattice is not unlimited, larger rare earth ions have a limited solubility in the spinel structure. XRD patterns of the S._01_, S_.03_, FS_.01_ and FS_.03_ samples are presented in Fig. 4b. As can be seen, coating of the nanoparticles with CA and F127 did not affect the positions of the diffraction peaks. However, the intensity of the peaks decreased which may be attributed to a lower crystallinity due to the presence of the ligands on the surface of the nanoparticles. Figure 5 shows some typical Rietveld-refined XRD patterns of as-prepared samples. As can be seen, the experimental and standard data were matched very well and no unwanted phases were observed. In the Rietveld analysis, the inverse spinel has been applied. It was purposed that zinc ions were in the A sites and samarium ions were in the B sites. Mean crystallite sizes, lattice constants of the samples, refinement parameters and quality of the patterns, such as goodness of fit (Sig), weighted profile R-factor (R_wp_), Bragg R-factor (R_b_) and the expected R-factor (R_exp_) are tabulated in Table 1.

**Table 1:** The lattice constants, the mean crystallite sizes and Rietveld-refined XRD data of the as-prepared Sm^3+^ substituted nanoparticles.

Sample	a ± 0.001(Å)	D ± 2(nm)	Sig (GoF)	R_wp_ (%)	R_b_ (%)	R_exp_ (%)
S._00_	8.370	26	0.55	26.48	16.80	48.2
S._01_	8.402	25	0.56	28.08	19.40	49.77
S._02_	8.426	24	0.55	26.48	16.80	48.2
S._03_	8.413	29	0.58	24.81	16.60	42.90
S._04_	8.410	30	0.58	24.15	16.57	41.72
S._05_	8.406	30	0.61	25.12	17.95	41.16

**Figure 6:**
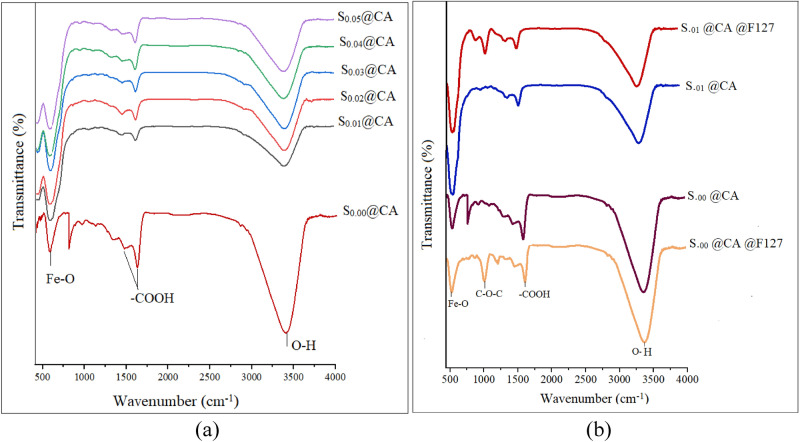
(**a**) FTIR spectra of Sm^3+^ and Zn^2+^ co- substituted magnetite nanoparticles coated with CA and (**b**) FTIR spectra of S_.00_ and S_.10_ samples coated with CA and CA@F127, i.e., FS_.00_ and FS_0.01_, respectively.

FTIR spectra of Zn_0.1_Sm_x_Fe_2.9-x_O_4_ nanoparticles coated with CA are illustrated in Fig. 6a. The broad band spectrum around 3411 cm^−1^ is attributed to the O–H band groups of water absorbed on the surface of nanoparticles. The band around 2900 cm^−1^ is related to the stretching vibration mode of the C–H bond. The 1720 cm^−1^ peak is due to the symmetric C=O stretching vibration mode from the –COOH group of CA which shifted to a lower wavenumber 1622 cm^−1^. The band around 1622 cm^−1^ is assigned to the binding of CA radicals on the surface of nanoparticles through the chemisorption of carboxylate citrate ions. The band around 1461 cm^−1^ is a characteristic band of the asymmetric stretching vibration mode of CO from the carboxylic group^39^. The intense band observed around 570 cm^−1^ in all FTIR spectra is assigned to stretching vibrational mode of Fe–O bonds on the tetrahedral and octahedral sites^40^. As can be seen, by increasing the Sm^3+^ content, this band shifted to lower wavenumbers which is attributed to the substitution by large Sm^3+^ on the octahedral site which affected distances of Fe–O bonds on the octahedral sites. Therefore, the bands were ascribed to the CA-coated MNPs. CA binds to the surface of nanoparticles through the carboxylate complex with the surface Fe ions. Figure 6b shows the FTIR spectra of coated S_.00_ and S_.01_ samples with both CA and Pluronic F127, i.e., FS_.00_ and FS_0.01_, respectively. The band around 1100 cm^−1^ is attributed to C–O–C stretching vibration mode of the PPO/PPE chains of pluronic F127^41^. The presence of this band confirms the adhesion of pluronic F127 on the surface of nanoparticles. It was reported that the intensity and the position of the band are composition dependent^42^.

**Figure 7:**
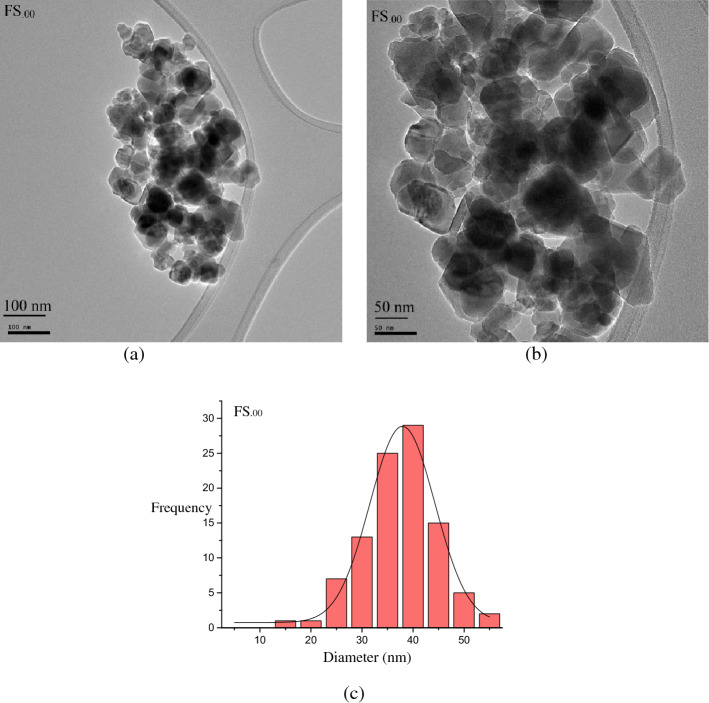
(**a**) and (**b**) TEM images of the FS_.00_ sample for 2 different magnifications and (**c**) corresponding histogram of the particle size distribution.

Figure 7a and b show TEM images of FS_.00_ nanoparticles for two different magnifications. As can be seen, nearly hexagonal and irregularly shaped were formed. Figure 7c shows the histogram of particle size distribution for the FS_.00_ sample, yielding an average of 40 nm. As the thicknesses of their shells were not clearly observable, we did not consider its thickness in averaging.

### Magnetic properties

**Figure 8:**
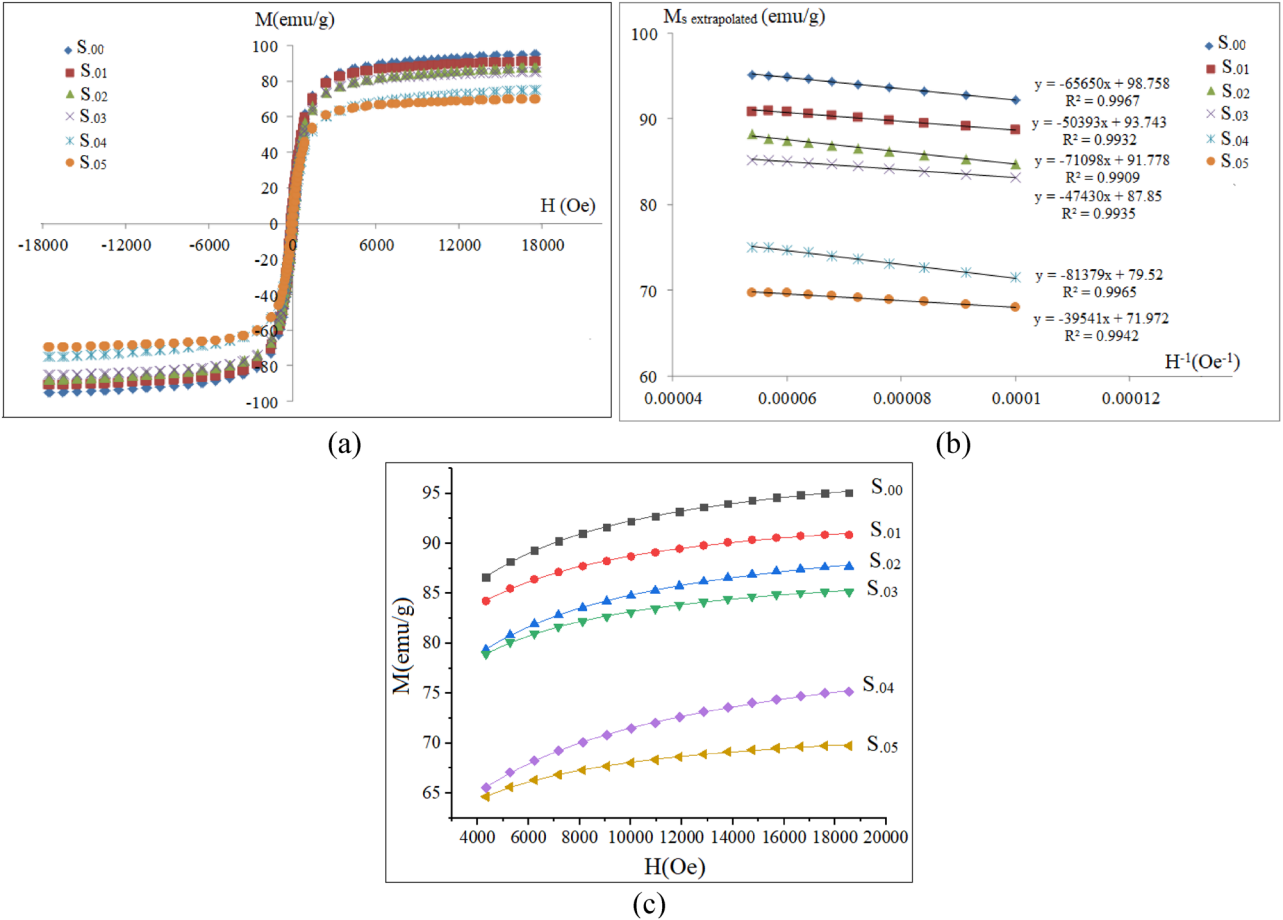
(**a**) Room temperature M-H curves of the as-prepared samples, before coating, (**b**) variation of M versus 1/H (for high field parts H > 10kOe) and linear fittings, and (**c**) fitting of the high field parts of the M-H curves, utilizing the law of approach to saturation.

The room temperature magnetization-magnetic field strength (M-H) loops and variations of magnetization as a function of 1/H (H > 10 kOe) as well as H curves for high fields are presented in Fig. 8a, b and c respectively. For all samples magnetization did not reach saturation at 18 kOe. Thus, the M-1/H curves were fitted linearly and extrapolated to (1/H → 0) to obtain saturation magnetization. The values of the magnetization at the maximum applied field (H = 18kOe), the extrapolated saturation magnetizations obtained from Fig. 8b, M_r_/M_s_ ratio, the estimated effective anisotropy constant K_eff_ utilizing the law of approach to saturation and the coercivity (H_c_) are given in Table 2. According to Néel theory for two sublattice ferrimagnetism, each sublattice is ordered ferromangetically and has a nonzero spontaneous magnetization and magnetizations of the two sublattices are coupled antiferromagnetically. Then, the magnitude of the net magnetization is obtained from:

3 $${\text{M}}({\text{T}}) = \left| {{\text{M}}_{{\text{A}}} ({\text{T}}) - {\text{M}}_{{\text{B}}} ({\text{T}})} \right|,$$

where M_A_, M_B_ and T are magnetizations of A, B sublattices and temperature, respectively. As can be seen, by increasing the content of Sm^3+^, the saturation magnetization gradually decreased which is due to replacement of Fe^3+^ (5 μ_B_) by Sm^3+^ (1.5 μ_B_) ions^43^ in B sites. Therefore, the number of magnetic moments of B sites was reduced, while the magnetization of the A sites remained constant. As can be seen, by increasing the content of Sm^3+^ the remanent to saturation magnetization ratio decreased slightly. According to the model of non-interacting randomly distributed uniaxial single domain particles, the M_r_/M_s_ ratio of the particles is 0.5. Any deviation from this value is indicating a transition to multidomain particles^44^. Table 2 illustrates that the M_r_/M_s_ ratios of the uncoated samples were less than 0.1, indicating that the particles are multidomains.

**Table 2:** The magnetic parameters of the uncoated samples.

Sample	M (@18kOe) (emu/g) ± 0.1	M_s extrapolated_ (emu/g) ± 0.1	M_r_/M_s_	H_c_(Oe) ± 0.1	K_eff_ × 10^3^ (erg/cm^3^)	T_c_ ± 5 °C
S_.00_	95.2	98.8	0.109	108.7	63.32	430
S_.01_	91.0	93.7	0.098	101.7	56.16	440
S_.02_	88.2	91.7	0.090	95.1	53.10	450
S_.03_	85.3	87.8	0.087	88.6	51.19	460
S_.04_	75.3	79.5	0.080	74.3	46.49	470
S_.05_	69.9	71.9	0.074	64.5	40.56	480

In order to estimate the effective anisotropy constants K_eff_ of the samples, an empirical equation, based on the law of approach to saturation, was used:

4 $${\text{M}}_{{\text{H}}} = {\text{M}}_{{\text{s}}} \left( {\text{T}} \right)\left[ {1 - \left( {\text{a/H}} \right) - \left( {{\text{b/H}}^{2} } \right)} \right] + {\text{cH}},$$

where M_H_ is magnetization component along the field direction and a, b and c are constants to be determined experimentally. The effective anisotropy constant K_eff_, can be calculated from K_eff_ = M_s_ (105b/8)^1/2^, in which b can be obtained from fitting of variation of M versus H near the saturation region, i. e., the high field (H > 4 kOe) part of the M-H curve^45,46^. Figure 8c shows the variation of magnetization versus magnetic field for high fields that was fitted to M_H_ = M_s_(T)[1-(a/H)-(b/H^2^)] + cH. All experimental data were fitted very well with R-squared values higher than 99%. As can be seen, by increasing the Sm^3+^ content the effective anisotropy constant K_eff_ decreased, which can be attributed to the effect of particle size. By increasing the Sm^3+^ content, a correlation between H_c_ and K_eff_ is observed, which is attributed to the direct relationship between H_c_ and K_eff_^46^:

5 $${\text{Hc}} = 0.64{\text{K}}_{{{\text{eff}}}} /{\text{M}}_{{\text{s}}} .$$

Table 2 illustrates that coercivity of the uncoated samples decreased as the Sm^3+^ content increased. Coercivity is dependent on the displacement of domain walls and on the particle sizes^7^. Coercivity also is impressed by other factors such as microstrain and magnetocrystalline anisotropy or interactions due to packing density, which is controlled from the coating^36,47^.

According to Berkov's theory for low anisotropy, coercivity first increases with increasing concentration when dipolar interaction becomes significance, but for more dense packing coercivity decreases again due to the formation of a short range order. Possibly, in the densely packed samples particles come locally in closer contact and therefore a coupling due to exchange interactions may become dominant, so some complicated magnetization structures arise which were not considered in Berkov's model^47^.

**Figure 9:**
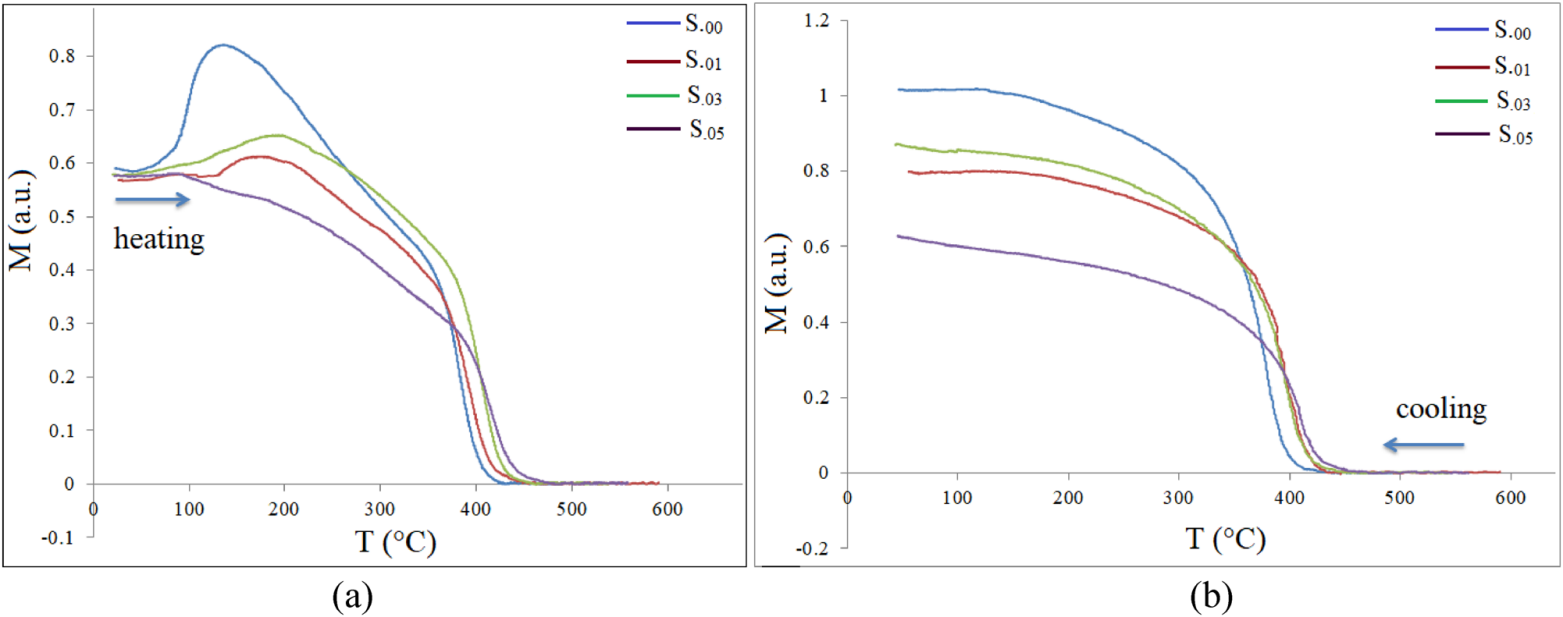
M-T curves of the samples, in heating (**a**) and in cooling (**b**) processes.

Figure 9 illustrates magnetization–temperature (M-T) curves of the as-prepared samples in the course of first heating and cooling processes. As can be seen, by increasing the Sm^3+^ content, the Curie temperature of the samples increased gradually from 430 °C to 480 °C. The Curie temperature is related to the number and the strength of magnetic interactions (A-A, B-B and A-B super-exchange interactions) as well as distance between paramagnetic ions^48^. In spinel ferrites, the interaction between Fe^3+^ ions in A and B sites is the strongest one and thus plays a dominant role in determining T_c_^7^. There is a weak exchange interaction between the 4f. electrons of Sm^3+^ and the 3d electrons of Fe^3+^, but A-O-B super-exchange interactions between Fe^3+^-Fe^3+^ is stronger than that of Fe^3+^-Sm^3+^. Furthermore, by increasing Sm^3+^ content, electron hopping between Fe^3+^ and Fe^2+^ ions in A and B sites occurs, which leads to a decrease in A-B super-exchange interaction^7^. Therefore, for a low concentration of Sm^3+^ ions, the lattice constant decreased as the Sm^3+^ content increased, A-B super-exchange interactions in the samples increased, all resulting in an increase in T_c_. As M-T curves were recorded in air, samples were oxidized during the determination of the Curie temperatures. The oxidation reaction of substituted magnetite (M_z_^2+^Fe_1-z_^2+^Fe_2_^3+^O_4_^2–^, where M^2+^ is a bivalent cation or a combination of cations with different valences so that their net valences are two or Fe^2+^Fe_2-x_^3+^M_x_^3+^O_4_^2−^, where M^3+^ is a trivalent cation or a combination of cations with net valence of three) is a topotactic reaction where the spinel structure is preserved. A metastable defect γ phase structure forms, which can be described by the following formula:

6 $$2\left( {{\text{M}}_{{\text{z}}}^{2 + } {\text{Fe}}_{{1 - {\text{z}}}}^{2 + } {\text{Fe}}_{2}^{3 + } {\text{O}}_{4}^{2 - } } \right) + 1/2{\text{O}}_{2} \to 3\gamma - \left( {{\text{Fe}}_{{2 - {\text{y}}}} {\text{M}}_{{2{\text{y}}/3}} {\text{O}}_{3} } \right),$$

where y = 4z/(9-z) and,

7 $$2\left( {{\text{Fe}}^{2 + } {\text{Fe}}_{{2 - {\text{x}}}}^{3 + } {\text{M}}_{{\text{x}}}^{3 + } {\text{O}}_{4}^{2 - } } \right) + 1/2{\text{O}}_{2} \to 3\gamma - \left( {{\text{Fe}}_{{2 - {\text{y}}}} {\text{M}}_{{\text{y}}} } \right)_{2} {\text{O}}_{3} ,$$

where y = x/3, 0 < y < 2/3.

By increasing the temperature, the cubic γ phase transforms into a stable rhombohedral hematite phase. Accordingly, the sample decomposes to a spinel and hematite structure^49–51^.

Solid solutions of γ-Fe_2_O_3_ and MFe_2_O_4_ are metastable and form during oxidation (< 600 °C) of substituted magnetite. During this process, high cation vacancy spinel ferrite forms^51^. As can be seen from M-T curves the magnetic ordering vanishes in the 430 to 480 °C temperature range and the samples showed paramagnetic behavior. It should mention that if the structure had a phase transition below 500 °C, different M-T curves should be observed. For more details refer to our previous work^29^.

During cooling from high temperatures to room temperature, magnetization showed a temperature dependency, which is due to the spinel phase with a new cation rearrangement based on ions preference energies which has ferrimagnetic order. The samples decomposed to hematite and a spinel phase. The Curie temperatures for all samples are summarized in Table 2.

### Colloidal properties

**Figure 10:**
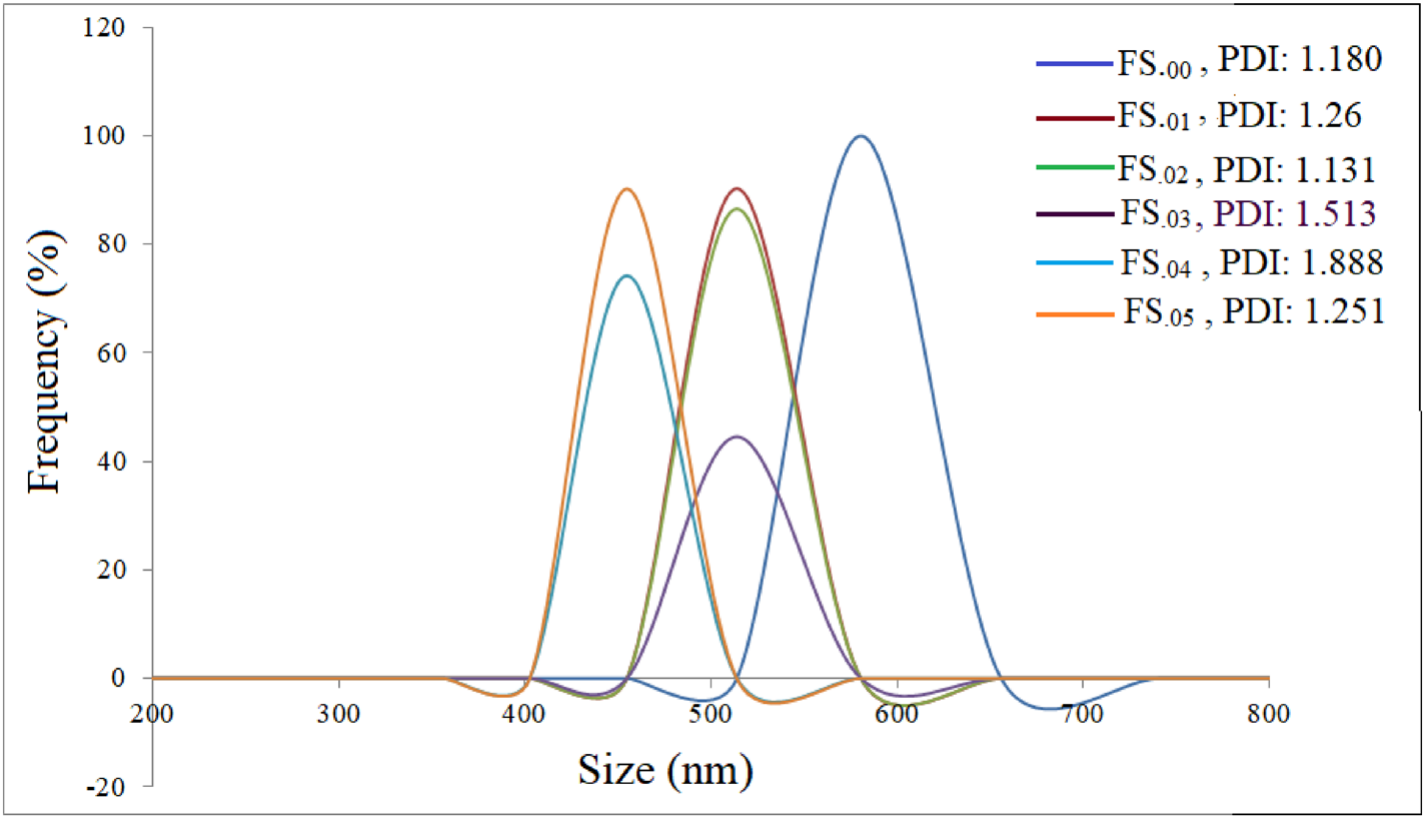
DLS diagrams of the ferrofluids, as marked on diagram.

Figure 10 shows DLS diagrams of the ferrofluids. The mean hydrodynamic sizes of the FS._00_, FS._01_, FS_.02_, FS_.03_, FS_.04_ and FS_.05_ coated nanoparticles, which were dispersed in an aqueous medium, at T = 25 °C and at pH = 5.5 were 582, 489, 491, 518, 445 and 433 nm, respectively. These sizes were much larger than those of both crystallite and particle, which indicates that the magnetic nanoparticles were agglomerated. The hydrodynamic size of the nanoparticles depends on their interactions and the numbers of polymers attached on their surfaces^52^. However, even at concentrations below the CMC, amphiphilic molecules attach onto the surfaces of nanoparticles, depending on their surface properties. For concentrations of the pluronic F127 in water above its CMC, micelles form. Possibly absence of micelles of pluronic F127 at T = 25 °C, the micelle passes its CMC, led to agglomeration and thus to a large hydrodynamic size.

### Calorimetrical and magnetic measurements on ferrofluids in an aqueous medium

To consider contributions of Néel and Brownian relaxation mechanisms for heat generation, we estimated the critical sizes as well as the effective anisotropy constant of the samples. For Néel relaxation, it is assumed that magnetic moments rotate, while the crystal structure is fixed in space. The Néel time constant is defined as:

8 $$\tau_{N} = \tau_{0} \exp \left( {{\text{K}}_{{{\text{eff}}}} {\text{V}}/{\text{k}}_{{\text{B}}} {\text{T}}} \right),$$

where τ_0_ = 10^−9^ s, k_B_ is the Boltzmann's constant (1.38 × 10^−23^ J/K), T is the absolute temperature, V is the particle volume and K_eff_ is effective magnetic anisotropy constant, which may originate from magnetocrystalline, shape and other anisotropies. For the Brownian relaxation mechanism, it is supposed that the magnetic moment is locked to the crystal structure. The magnetic moment rotates in a low viscosity carrier medium, when it aligns with the applied field.

The Brownian time constant is:

9 $$\tau_{{\text{B}}} = 3\eta {\text{V}}_{{\text{H}}} /{\text{k}}_{{\text{B}}} {\text{T}},$$

where η is the viscosity of the carrier liquid, which is 1.01 × 10^−3^ kg/sm for water and V_H_ is the hydrodynamic volume^53,54^.

In presence of a magnetic field varying in time, the Brownian relaxation mechanism results in a heat generation in a ferrofluid, as a consequence of viscous friction between particles and the surrounding carrier liquid, which is not limited to superparamagnetic particles^55^.

We presume that magnetocrystalline anisotropy prevails in effective magnetic anisotropy of the nanoparticles. At a high frequency, the critical size is defined by ωτ = 1^56^. In the critical size region the hysteresis loss vanishes abruptly and relaxation effects form remaining loss mechanisms^57^. The estimated effective anisotropy constants of the samples are given in Table 2. For the applied frequency (290 kHz) the critical particle size of the samples were found to be in the range from 19 to 22 nm, where p_Néel_ achieved its maximum. The produced nanoparticles have larger average sizes than the critical size. Therefore, high SLP values are due to nonzero coercivity, i. e., due to hysteresis loss. The hydrodynamic size for our measuring frequency was 11 nm for ωτ_B_ = 1. The hydrodynamic sizes of the prepared samples are marked in Fig. 10, which are very higher than those obtained from the hydrodynamic volumes (V_H_), estimated from Eq. (9).

**Figure 11:**
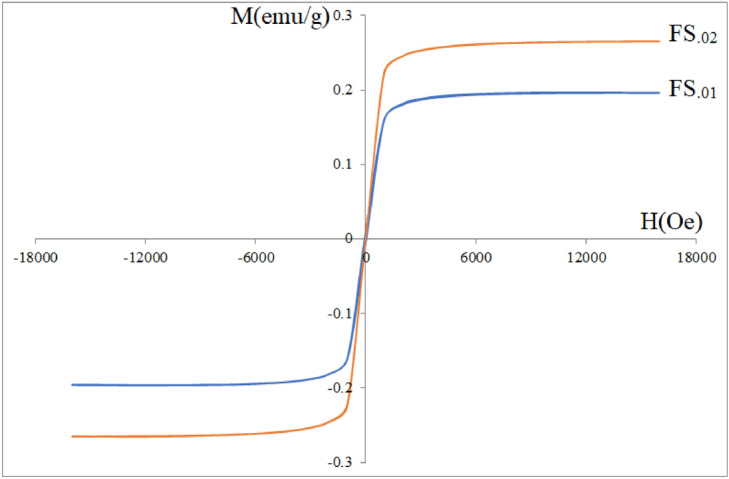
Room temperature M-H curves of the FS_.01_ and FS_.02_ in an aqueous medium.

**Figure 12:**
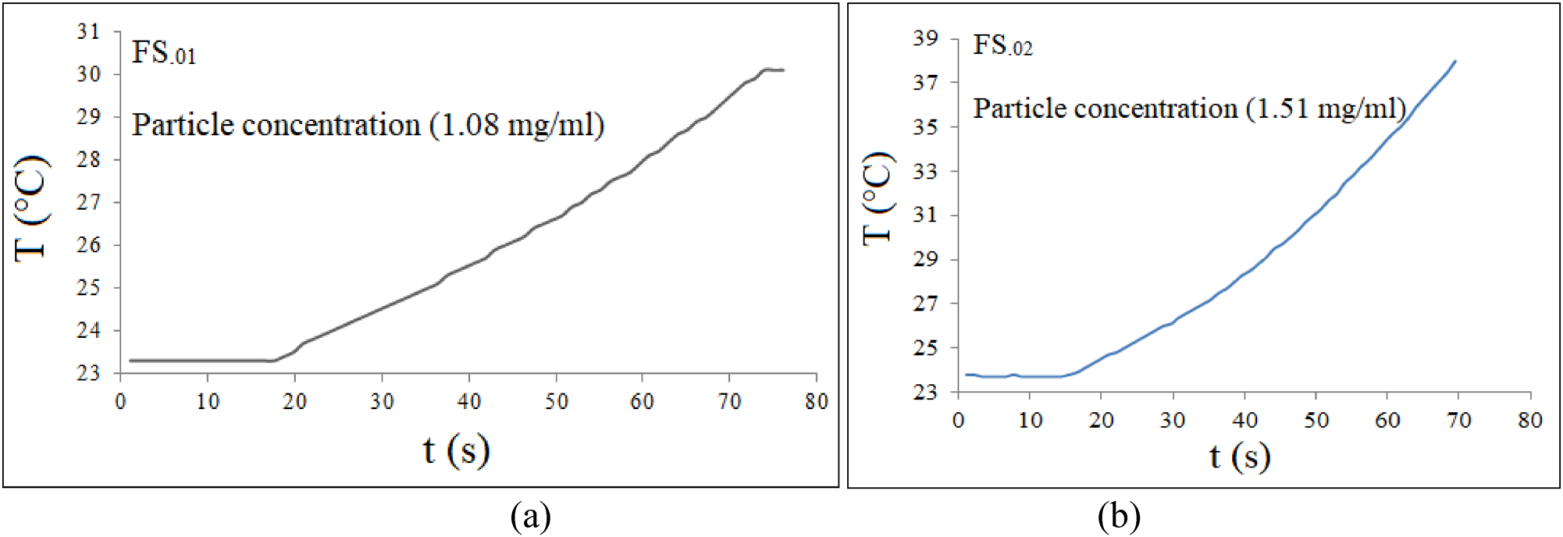
The variation of the temperature with respect to time of the liquid ferrofluids in an RF magnetic field.

Figure 11 shows room temperature VSM curves, which were carried out on the aqueous ferrofluids (FS_.01_ and FS_.02_). As can be seen although magnetizations of the samples were saturated, but they are so small, which is due to very low concentrations. Saturation magnetization of a ferrofluid in an aqueous medium not only depends on the single cores M_S_, but depends on concentration. Also VSM data reviled that both ferrofluids have moderate coercivities and then no superparamagnetic behavior, which is due to slightly agglomeration in aqueous medium. As discussed above the same agglomeration was observed by DLS measurements too. So we can deduce that relaxation processes did not play a dominant role in SLP. In an external RF magnetic field, magnetic materials convert the incident RF energy into heat^58^. The heating efficiency of the magnetic ferrofluids was obtained using SLP (W/g) = dT/dt × Σ m_i_C_i_/m. The calculated SLP and ILP values are summarized in Table 3. The maximum SLP and ILP were found to be 259 W/g and 3.49 nHm^2^/kg, respectively, for the sample with x = 0.01. The SLP depends on magnetic field parameters and properties of the nanoparticles, such as size, size distribution, saturation magnetization M_S_, remanent magnetization M_R_, coercivity H_C_ and effective magnetic anisotropy as well as the concentration of the sample. Hysteresis loss represents the main portion in heat generation of multidomain magnetic materials, while relaxation processes represent the main contribution for single domain superparamagnetic particles^17^. Therefore the high SLP values of the FS_.01_ and FS_.02_ samples can be attributed to hysteresis loss first and Brownian mechanism second, due to viscous friction between rotating particles and surrounding aqueous medium. Figure 12a and b illustrate the variation of the temperature in terms of time (T-t) curves for x = 0.01 and 0.02 in an RF magnetic field. According to data from Table 3 and the temperature variations (ΔT) with respect to time, the FS_.01_ and FS_.02_ could potentially be good candidates for magnetic hyperthermia application. Gadzhimagomedova et al. reported SLP and ILP values of 8.2 W/g and 0.15 nHm^2^/kg, respectively for samarium doped superparamagnetic magnetite nanoparticles (Sm_0.033_Fe_2.967_O_4_) coated with PEG^59^, which are very lower than those we obtained in this work, Table 3.

**Table 3:** Specific loss power (SLP) and intrinsic loss power (ILP) of the aqueous ferrofluids.

Sample1	FS_.00_	FS_.01_	FS_.02_	FS_.03_	FS_.04_	FS_.05_
SLP (W/g)	373	259	232	107	188	184
ILP (nHm^2^/kg)	5.02	3.49	3.13	1.44	2.53	2.44

## Conclusions

Single phase Sm^3+^ and Zn^2+^ co-substituted magnetite (Zn_0.1_Sm_x_Fe_2.9-x_O_4_, x = 0.01, 0.02, 0.03, 0.04 and 0.05) nanoparticles were synthesized via co-precipitation method. The comparatively large (d > 20 nm) nanoparticles were coated with citric acid and pluronic F127, using a simple route to obtain core–shell structures and then suspended in water to get stable ferrofluids. The stability of the ferrofluids (pH 5.5) was related to the formation of micelles. The PPO sequence of the pluronic F127 adsorbed on the surface of the nanoparticles, while the PEO chains formed hydrophilic shells around the nanoparticles, which in turn generated repulsive forces due to entropy reasons. The coercivity, (M_r_/M_s_) ratio and the effective anisotropy constant K_eff_ were monotonically decreased by increasing the Sm^3+^ content, which related to properties of the nanoparticles, such as size, size distribution and morphology. VSM measurements on all ferrofluids presented nonzero coercivities, which are results of both multidomain cores and agglomeration of the coated nanoparticles in the solution. The highest obtained values of the SLP and ILP were 259 W/g and 3.49 nHm^2^/kg, respectively, which were found for the FS_.01_ sample at a concentration as low as 1.08 mg/ml. Those high values are due to the nonzero coercivity of the samples that leads to a hysteresis loss portion as the main loss mechanism, although frictional loss may have occurred additionally too. From the SLP and ILP data, we concluded that both FS_.01_ and/or FS_.02_ samples are good candidates for magnetic hyperthermia applications potentially to achieve sufficient heating efficiencies at a low magnetic nanoparticles concentration.

## References

[1] Tomitaka A, Ueda K, Yamada T, Takemura Y (2012). Heat dissipation and magnetic properties of surface-coated Fe_3_O_4_ nanoparticles for biomedical applications. J. Magn. Magn. Mater..

[2] Dutz S, Buske N, Landers J, Grafe C, Wende H, Clement JH (2020). Biocompatible magnetic fluids of Co-doped iron oxide nanoparticles with tunable magnetic properties. Nanomaterials.

[3] Shaterabadi Z, Nabiyouni G, Soleymani M (2017). High impact of in situ dextran coating on biocompatibility, stability and magnetic properties of iron oxide nanoparticles. Mater. Sci. Eng., C.

[4] Srinivasamurthy KM, Angadi VJ, Kubrin SP, Matteppanavar S, Kumar PM, Rudraswamy B (2018). Evidence of enhanced ferromagnetic nature and hyperfine interaction studies of Ce-Sm doped Co-Ni ferrite nanoparticles for microphone applications. Ceram. Int..

[5] Phong PT, Nguyen LH, Phong LTH, Nam PH, Manh DH, Lee IJ, Phuc NX (2017). Study of specific loss power of magnetic fluids with various viscosities. J. Magn. Magn. Mater..

[6] Naik PP, Tangsal RB, Meena SS, Yusuf SM (2017). Influence of rare earth (Nd^3+^) doping on structural and magnetic properties of nanocrystalline manganese-zinc ferrite. J. Mater. Chem. Phys..

[7] Liu Z, Peng Zh, Lv Ch, Fu X (2017). Doping effect of Sm^3+^ on magnetic and dielectric properties of Ni-Zn ferrites. J. Ceram. Int..

[8] Jiang J, Li L, Xu F, Xie Y (2007). Preparation and magnetic properties of Zn–Cu–Cr–Sm ferrite via a rheological phase reaction method. J. Mater. Sci. Eng. B.

[9] Polozhentsev OE, Kubrin SP, Butova VV, Kochkina VK, Soldatov AV, Stashenko VV (2017). Structure and magnetic properties of pure and samarium doped magnetite nanoparticles. J. Struct. Chem..

[10] Thankachan S, Jacob BP, Xavier S, Mohammed EM (2013). Effect of samarium substitution on structural and magnetic properties of magnesium ferrite nanoparticles. J. Magn. Magn. Mater..

[11] Yadav N, Kumar A, Rana PS, Rana DS, Arora M, Pant RP (2015). Finite size effect on Sm^3+^ doped Mn_0.5_Zn_0.5_Sm_x_Fe_2−x_O_4_ (0 ≤ x ≤ 0.5) ferrite nanoparticles. Ceram. Int..

[12] Nabeel AI (2020). Samarium enriches antitumor activity of ZnO nanoparticles via downregulation of CXCR4 receptor and cytochrome P450. Tumor Biology.

[13] Hejase H, Hayek SS, Qadri S, Haik Y (2012). MnZnFe nanoparticles for self-controlled magnetic hyperthermia. J. Magn. Magn. Mater..

[14] Tang YD, Flesh RCC, Zhang C, Jin T (2018). Numerical analysis of the effect of non-uniformity of the magnetic field produced by a solenoid on temperature distribution during magnetic hyperthermia. J. Magn. Magn. Mater..

[15] Robles J, Das R, Glassell M, Phan MH, Srikanth H (2018). Exchange-coupled Fe_3_O_4_/CoFe_2_O_4_ nanoparticles for advanced magnetic hyperthermia. J. AIP Adv..

[16] Reena Mary AP, Narayanan TN, Sunny V, Sakthikumar D, Yoshida Y, Joy PA, Anantharaman MR (2010). Synthesis of bio-compatible spion-based aqueous ferrofluids and evaluation of radiofrequency power loss for magnetic hyperthermia. J. Nanoscale Res. Lett..

[17] Dutz S, Hergt R (2014). Magnetic particle hyperthermia—a promising tumour therapy?. Nanotechnology.

[18] Campelj S, Makovec D, Drofenik M (2008). Preparation and properties of water-based magnetic fluids. J. Phys. Condens. Matter Inst. Phys. J..

[19] de Sousa ME, FernándezvanRaap MB, Rivas PC, MendozaZélis P, Girardin P, Pasquevich GA, Alessandrini JL, Muraca D, Sánchez FH (2013). Stability and relaxation mechanisms of citric acid coated magnetite nanoparticles for magnetic hyperthermia. J. Phys. Chem. C.

[20] Chander V, Gangenahalli G (2020). Pluronic-F127/platelet microvesicles nanocomplex delivers stem cells in high doses to the bone marrow and confers post-irradiation survival. J. Sci. Rep..

[21] Alexandridis P, Alan Hatton T (1995). Poly(ethylene oxide)-poly(propylene oxide )-poly (ethylene oxide) block copolymer surfactants in aqueous solutions and at interfaces: thermodynamics, structure, dynamics, and modeling. Colloids Surf. A Physicochem. Eng..

[22] Reyes-Rodríguez PY, Cortés-Hernández DA, Ávila-Orta CA, Sánchez J, Andrade-Guel M, Herrera-Guerrero A, Cabello-Alvarado C, Ramos-Martínez VH (2021). Synthesis of Pluronic F127-coated magnesium/calcium (Mg_1-x_Ca_x_Fe_2_O_4_) magnetic nanoparticles for biomedical applications. J. Magn. Magn. Mater..

[23] Manaspon C, Viravaidya-Pasuwat K, Pimpha N (2012). Preparation of folate-conjugated Pluronic F127/chitosan core-shell nanoparticles encapsulating doxorubicin for breast cancer treatment. J. Nanomater..

[24] Zargar T, Kermanpur A (2017). Effects of hydrothermal process parameters on the physical, magnetic and thermal properties of Zn_0.3_Fe_2.7_O_4_ nanoparticles for magnetic hyperthermia applications. Ceram. Int..

[25] Dutz S, Clement JH, Eberbeck D, Gelbrich T, Hergt R, Müller R, Wotschadlo J, Zeisberger M (2009). Ferrofluids of magnetic multicore nanoparticles for biomedical applications. J. Magn. Magn. Mater..

[26] Li X, Park EK, Hyun K, Oktavia L, Kwak M (2018). Rheological analysis of core-stabilized Pluronic F127 by semi-interpenetrating network (SIPN) in aqueous solution. Soc. Rheol..

[27] Cullity BD (1956). Elements of X-Ray Diffraction.

[28] Mozaffari M, NováK C, Pokol G, Sztaniszláv A (2002). Semi-quantitative determination of magnetic phase composition in YIG using TG (M) method. J. Electr. Eng..

[29] Shatooti S, Mozaffari M (2019). The effect of Zn^2+^ substitution on magnetic properties of maghemite nanoparticles, prepared by one-pot coprecipitation method at room temperature. J. Mater. Sci.: Mater. Electron..

[30] Dutz S, Muller R, Eberbeck D, Hilger I, Zeisberger M (2015). Magnetic nanoparticles adapted for specific biomedical applications. J. Biomed. Tech. Biomed Eng..

[31] Jordan A, Wust P, Fähing H, Johns W, Hinz A, Felix R (1993). Inductive heating of ferrimagnetic particles and magnetic fluids: physical evaluation of their potential for hyperthermia. Int. J. Hyperth..

[32] Behdadfar B, Kermanpur A, Sadeghi-Aliabadi H, Morales MDP, Mozaffari M (2012). Synthesis of aqueous ferrofluids of Zn_x_Fe_3−x_O_4_ nanoparticles by citric acid assisted hydrothermal-reduction route for magnetic hyperthermia applications. J. Magn. Magn. Mater..

[33] Denton AR, Aschcroft NW (1991). Vegard’s law. Phys. Rev. A.

[34] Ismail SM, Yehia M, Ata-Allah SS (2015). Influence of zinc doping on the structural and magnetic properties of Ni-Ga-Sm polycrystalline ferrites. J. Supercond. Novel Magn..

[35] Karimi S, Kameli P, Ahmadvand H, Salamati H (2016). Effects of Zn-Cr-substitution on the structural and magnetic properties of Ni_1−x_Zn_x_Fe_2−x_Cr_x_O_4_ ferrites. Ceram. Int..

[36] Jasrotia R, Suman AA, Singh VP, Kumar R, Verma R, Chauhan A (2019). Effect of Y^3+^, Sm^3+^ and Dy^3+^ ions on the microstructure, morphology, optical and magnetic properties NiCoZn magnetic nanoparticles. Results Phys..

[37] Qin W, Nagase T, Umakoshi Y, Szpunar JA (2008). Relationship between microstrain and lattice parameter change in nanocrystalline materials. Philos. Mag. Lett..

[38] Yehia M, Ismail SM, Hashhash A (2014). Structural and magnetic studies of rare-earth substituted nickel ferrites. J. Supercond. Novel Magn..

[39] Dheyab MA, Aziz AA, Jameel MS, Noqta OA, Khaniabadi PM, Mehrdel B (2020). Simple rapid stabilization method through citric acid modification for magnetite nanoparticles. Sci. Rep..

[40] Nasrazadani S, Raman A (1993). The application of infrared spectroscopy to the study of rust system–II. study of cation deficiency in magnetite (Fe_3_O_4_) producedduring its transformation to maghemite (γ-Fe_2_O_3_) and hematite (α-Fe_2_O_3_). Corros. Sci..

[41] Jain TK, Foy SP, Erokwu B, Dimitrijevic S, Flask CA, Labhasetwar V (2009). Magnetic resonance imaging of multifunctional pluronic stabilized iron-oxide nanoparticles in tumor-bearing mice. Biomaterials.

[42] El Hiti MA, El Shora AI, Seoud AS, Hammad SM (2006). Structural studies for Zn_x_Mg_0.8-x_Ni_0.2_Fe_2_O_4_ ferrites. Phase Trans..

[43] Wilkinson G, Gillard RD, McCleverty JA (1987). Comprehensive Coordination Chemistry, Volume I.

[44] Luna C, del Puerto Morales M, Serna CJ, Vazquez M (2003). Multidomain to single-domain transition for uniform Co_80_Ni_20_ nanoparticles. Nanotechnology.

[45] Wang L, Rai BK, Mishra SR (2015). Structural and magnetic study of Al^3+^ doped Ni_0.75_Zn_0.25_Fe_2-x_Al_x_O_4_ nanoferrites. Mater. Res. Bull..

[46] Morrish, A. H. *The Physical Principles of Magnetism* (The Institute of Electrical and Electronics Engineers, Inc., 2001).

[47] Dutz S, Hergt R (2012). The role of interactions in systems of magnetic iron oxide nanoparticles in the single domain size range. J. Nano- Electron. Phys..

[48] Mozaffari M (2015). Synthesis of Zn^2+^ substituted maghemite nanoparticles and investigation of their structural and magnetic properties. J. Magn. Magn. Mater..

[49] Khan Y, Kneler E (1978). Structre and magnetic moment of zinc-substituted γ iron oxide. J. Magn. Magn. Mater..

[50] Gillot B, Chassagneux F, Rousset A (1981). Oxidation in the γ phase of spinels containing iron II: influence of defects on the oxidation kinetics and electrical properties. J. Solid State Chem..

[51] Kodama T (1992). High-vacancy-content zinc (II)-bearing ferrites from iron (III) tartrate in strongly alkaline solutions. J. Mater. Chem..

[52] Zargar T, Kermanpur A, Labbaf S, Houreh AB, Esfahani MHN (2018). PEG coated Zn_0.3_Fe_2.7_O_4_ nanoparticles in the presence of α-Fe_2_O_3_ phase synthesized by citric acid assisted hydrothermal reduction process for magnetic hyperthermia applications. Mater. Chem. Phys..

[53] Rosensweig RE (2002). Heating magnetic fluid with alternating magnetic field. J. Magn. Magn. Mater..

[54] Dutz S, Hergt R, Mürbe J, Müller R, Zeisberger M, Andrä W, Töpfer J, Bellemann ME (2007). Hysteresis losses of magnetic nanoparticle powders in the single domain size range. J. Magn. Magn. Mater..

[55] Hergt R, Dutz S, Müller R, Zeisberger M (2006). Magnetic particle hyperthermia: nanoparticle magnetism and materials development for cancer therapy. J. Phys.: Condens. Matter.

[56] Hergt R, Andrä W, d’Ambly CG, Hilger I, Kaiser WA, Richter U, Schmidt HG (1998). Physical limits of hyperthermia using magnetite fine particles. IEEE Trans. Magn..

[57] Behdadfar B, Kermanpur A, Sadeghi-Aliabadi H, Morales MDP, Mozaffari M (2012). Synthesis of high intrinsic loss power aqueous ferrofluids of iron oxide nanoparticles by citric acid-assisted hydrothermal-reduction route. J. Solid State Chem..

[58] Shaterabadi Z, Nabiyouni G, Soleymani M (2018). Physics responsible for heating efficiency and self-controlled temperature rise of magnetic nanoparticles in magnetic hyperthermia therapy. Prog. Biophys. Mol. Biol..

[59] Gadzhimagomedova ZM, Polozhentsev OE, Kuchma EA, Soldatov MA, Kirsanova DYu, Maximov AYu, Soldatov AV (2020). Local atomic and electronic structures of superparamagnetic nanoparticles based on iron oxides for local hyperthermia in oncology. Nanotechnol. Russ..

